# D-Pinitol Mitigates Renal Senescence via Targeting the SARM1-cGAS-STING Signaling Axis to Restore Mitochondrial Function and Dampen Inflammatory Responses

**DOI:** 10.3390/biomedicines14051092

**Published:** 2026-05-12

**Authors:** Xiaofan Yin, Kaizhi Wen, Kena Yu, Zhengxin Liu, Weiming He

**Affiliations:** 1 First School of Clinical Medicine, Nanjing University of Chinese Medicine, Nanjing 210023, China; 20230054@njucm.edu.cn (X.Y.); 13290558270wkz@sina.com (K.W.); janice1989@126.com (K.Y.); lxinguangzx@163.com (Z.L.); 2 Department of Nephrology, Affiliated Hospital of Nanjing University of Chinese Medicine, Nanjing 210029, China

**Keywords:** D-Pinitol, SARM1, mitochondrion, cGAS-STING pathway, renal aging

## Abstract

**Background**: Renal aging represents a pivotal contributor to the pathogenesis and progression of age-related kidney disorders. D-Pinitol (DP), a bioactive cyclitol naturally present in food plants, exhibits multiple beneficial biological activities. Nevertheless, its role in counteracting renal aging remains unclear. **Methods**: This study employed both in vitro (HK-2 cells) and in vivo (C57BL/6J mice) models of D-galactose (DG)-induced renal aging. A panel of experimental approaches was applied to characterize the protective effects and molecular mechanisms of DP against renal aging, including Western blot, qPCR, ELISA, transcriptomic profiling, transmission electron microscopy, surface plasmon resonance (SPR), immunohistochemistry, and immunofluorescence staining. **Results**: DP significantly attenuated DG-induced renal aging-like changes in vitro and in vivo by preserving mitochondrial function and alleviating inflammatory responses. Transcriptomic analysis suggested SARM1 as a potential key target responsible for the beneficial effects of DP. In DG-induced aging models, SARM1 was remarkably upregulated in a tubule-specific pattern and acted as a critical mediator of mitochondrial dysfunction. Damaged mitochondria released mtDNA, which further activated the cGAS–STING innate immune signaling pathway, consequently promoting the senescence-associated secretory phenotype (SASP) and renal inflammation. Mechanistically, molecular docking and related assays suggested that DP may stabilize the auto-inhibitory conformation of SARM1, thereby potentially preventing its activation. **Conclusions**: DP attenuates DG-induced renal aging-like changes via suppressing the SARM1–cGAS–STING axis, thereby restoring mitochondrial homeostasis and mitigating inflammation. Given the lack of effective interventions targeting renal aging, these findings suggest SARM1 as a novel potential therapeutic target for renal aging and highlight DP as a promising food-derived anti-aging ingredient for renal protection.

## 1. Introduction

Global population aging stands as one of the most profound socio-demographic shifts in the 21st century. This demographic transition has driven a sharp surge in the prevalence and burden of age-related chronic conditions, creating substantial pressures on global socioeconomic frameworks and healthcare infrastructure [1]. Against this backdrop, chronic kidney disease (CKD) has seen a sustained rise in global prevalence, emerging as a pressing worldwide public health concern. Projections indicate that CKD will become the fifth leading cause of death globally by 2050, with even higher rankings—second or third place—expected in long-lifespan nations such as Japan and Spain. Critically, the projected 33.1% global rise in age-adjusted CKD mortality is vastly outpaced by the 140% increase in all-age all-cause mortality, a discrepancy that confirms population aging as the primary driver of the growing CKD burden [2].

Emerging evidence has demonstrated that, across all chronic non-communicable diseases, CKD exhibits the most robust correlation with accelerated individual biological aging. On average, patients with CKD have a biological age 6.15 years higher than their chronological age, with their renal organs showing an even more pronounced 8.03-year acceleration in biological aging [3]. Consequently, premature renal aging is a significant risk factor for the onset and progression of CKD. Cellular senescence is considered a key mechanism driving renal aging [4]. Senescent cells undergo irreversible cell cycle arrest, and concurrently secrete a broad array of inflammatory cytokines, chemokines, growth factors, and proteases—a molecular profile defined as the senescence-associated secretory phenotype (SASP) [5]. Via autocrine and paracrine signaling cascades, SASP establishes a persistently pro-inflammatory and pro-fibrotic tissue microenvironment. This pathological state not only worsens functional impairment in the senescent cells themselves, but also drives senescence in adjacent healthy cells, forming a self-reinforcing vicious cycle that ultimately results in tissue damage and progressive organ functional deterioration [6]. Therefore, targeted suppression of SASP represents a critical interventional strategy to delay renal aging and halt CKD progression.

The D-galactose (DG)-induced subacute aging model is a well-established preclinical system widely utilized in renal aging investigations [7,8]. The broad applicability of this model arises from its capacity to faithfully recapitulate the core molecular and cellular drivers of physiological aging in a systemic manner [9]. Exposure to high-dose DG triggers robust oxidative stress and activates pro-inflammatory signaling cascades including the NF-κB pathway, which in turn drives robust SASP factor secretion. These characteristics enable researchers to observe hallmark pathological features of renal aging within a compressed experimental timeframe, including cellular senescence, SASP secretion, mitochondrial dysfunction and inflammatory response [10]. Hence, this model was selected for the present study to evaluate anti-aging interventions targeting SASP release.

D-Pinitol (3-O-methyl-D-chiro-inositol, DP) is a naturally occurring bioactive cyclitol, abundant in edible legumes and pine-derived plant sources [11]. Accumulating preclinical evidence has highlighted the promising renal protective potential of DP. Its well-characterized antioxidant and anti-inflammatory properties have been validated across multiple preclinical models of renal injury, including those induced by cyclosporine A, cisplatin, and streptozotocin [12,13,14]. Furthermore, prior work has also shown that DP supplementation enhances antioxidant defense capacity and extends lifespan in Caenorhabditis elegans models [15]. Despite these findings, several important gaps remain in the research on D-Pinitol. Existing studies on D-Pinitol in the kidney have focused mainly on acute injury or diabetic nephropathy, while its role in renal aging has never been explored. Moreover, although D-Pinitol is known to possess anti-inflammatory and antioxidant activities, the upstream signaling mechanisms that link these effects to the suppression of the senescence-associated secretory phenotype (SASP) have not been investigated. Based on the renal protective effects and anti-aging potential of DP, we hypothesized that DP might delay renal aging. To verify this hypothesis, we systematically evaluated the protective efficacy of DP against renal aging, and dissected the underlying molecular mechanisms, using complementary in vitro and in vivo experimental systems.

## 2. Materials and Methods

### 2.1. Animal Experiments

All animal procedures were approved by the Animal Ethics Committee of Nanjing University of Chinese Medicine (Approval No. 202411A022). Six- to eight-week-old male SPF-grade C57BL/6J mice were obtained from Jiangsu Huachuang Xinnuo Pharmaceutical Technology Co., Ltd. (Taizhou, China), and housed under a 24 ± 1 °C environment with a 12 h light/dark cycle, with ad libitum access to standard chow and sterile water. After 7-day acclimation, mice were randomly allocated into 5 groups (*n* = 6 per group): control group, D-galactose (DG) model group, low-dose DP (DPL) group, high-dose DP (DPH) group, and vitamin E (VE) positive control group.

The control group received daily subcutaneous injection of equal-volume normal saline; the DG model group was given daily subcutaneous injection of 250 mg/kg DG (#A600215-0100, BBI, Shanghai, China) dissolved in saline [7,16]. The DPL and DPH groups received identical DG injection plus daily oral gavage of 50 mg/kg/d and 100 mg/kg/d DP (#HY-N0655, MedChemExpress, Monmouth Junction, NJ, USA) in double-distilled water, respectively; the VE group was given DG injection plus daily oral gavage of 100 mg/kg/d VE dissolved in sesame oil. These doses were selected based on previous literature showing a safe and effective range of 50–150 mg/kg/d for DP in rodent models [12,13,14]. All interventions lasted for 6 weeks, with dosing volumes adjusted weekly based on body weight.

At the end of the experiment, mice were anesthetized with Zoletil 50 for sample collection. Bilateral kidneys were harvested: one was fixed in 4% paraformaldehyde for histological analysis, a portion of the contralateral kidney was processed for transmission electron microscopy (TEM, HITACHI, Tokyo, Japan), and the remaining renal tissue was stored at −80 °C for subsequent experiments.

### 2.2. Cell Culture

The human renal proximal tubular epithelial cell line HK-2 was obtained from Wuhan Procell Life Technology Co., Ltd. (Wuhan, China). Cells were maintained in DMEM/F12 complete medium (#L310KJ, BasalMedia, Shanghai, China), supplemented with 10% fetal bovine serum (FBS; #10099141C, Gibco, Waltham, MA, USA) and 1% 100× penicillin-streptomycin solution (#S110JV, BasalMedia). All cell cultures were incubated in a humidified 5% CO_2_ atmosphere at a constant 37 °C. Culture medium was refreshed every 2 to 3 days. When cell confluency reached 80–90%, passaging was performed using 0.25% trypsin-EDTA solution (#S310KJ, BasalMedia).

### 2.3. Plasmid and siRNA Transfection

Cell transfection was conducted when cells reached 40–60% confluency. Lipofectamine™ 3000 transfection reagent (#L3000075, Thermo Fisher Scientific, Waltham, MA, USA) and the required siRNA or plasmid DNA were diluted separately in Opti-MEM medium (#L531KJ, BasalMedia). For plasmid transfection, P3000™ Enhancer was supplemented to the diluted plasmid solution, while this step was omitted for siRNA transfection. The diluted transfection reagent was subsequently mixed with the diluted siRNA or plasmid mixture, followed by a 15-min incubation at room temperature to allow the formation of transfection complexes. After incubation, the prepared complexes were added dropwise to cells cultured in antibiotic-free medium. At 8 h after transfection, the medium was replaced with fresh complete medium, and cells were harvested for subsequent experimental analysis at 48 h post-transfection. All plasmids and siRNA sequences used in this study were synthesized and provided by Shanghai GenePharma Co., Ltd. (Shanghai, China).

### 2.4. Cell Viability Assay

Cell viability was evaluated via the Cell Counting Kit-8 (CCK-8) assay, strictly following the manufacturer’s standard protocol. Briefly, after the designated experimental treatments, 100 µL of complete medium supplemented with 10% CCK-8 reagent (#C0037, Beyotime Biotechnology, Shanghai, China) was added to each well of the culture plate. The plates were subsequently incubated for 1 h in a light-proof environment at 37 °C. The absorbance value at 450 nm was detected using a Synergy H1M2 microplate reader (#Synergy H1M2, BioTek Instruments, Winooski, VT, USA), with the measured values used to reflect the relative cell viability of each group.

### 2.5. HE and SA-β-gal Staining

Following 24 h of fixation, harvested renal tissues were trimmed, rinsed thoroughly, and processed through sequential dehydration in graded ethanol solutions, followed by clearing in xylene and paraffin embedding. The embedded tissue blocks were sectioned at a thickness of 4 µm, floated on warm deionized water, mounted onto adhesive glass slides, and baked at 60 °C for subsequent staining. The sections were then deparaffinized, rehydrated through a gradient ethanol series, and stained with Harris Hematoxylin and Eosin (HE) according to standard histological protocols. All reagents used for HE staining were obtained from Hubei BIOSSCI Biotechnology Co., Ltd. (Wuhan, China).

SA-β-gal staining was performed using the Senescence β-Galactosidase Staining Kit (#C0602, Beyotime). Mouse kidney tissues were embedded in OCT compound, frozen in liquid nitrogen, and sectioned (20 µm). Rewarmed sections or HK-2 cells were fixed with β-galactosidase staining fixative (room temperature, 15 min), washed with PBS (tissues: 3 × 5 min; cells: 3 × 3 min), and incubated with staining working solution (pH 6.0) at 37 °C overnight (16–18 h) in a dry incubator (without CO_2_). Blue-stained cells were counted as senescent cells.

### 2.6. Western Blot Analysis

Total protein was extracted from harvested renal tissues and HK-2 cells using RIPA lysis buffer (#P0013B, Beyotime), freshly supplemented with 1× protease and phosphatase inhibitor cocktail (#P1045, Beyotime) and phenylmethylsulfonyl fluoride (PMSF; #ST505, Beyotime). Protein concentration of each sample was quantified via the BCA method using the Pierce BCA Protein Assay Kit (#23227, Thermo Fisher Scientific).

Approximately 30 µg of total protein per sample was separated by sodium dodecyl sulfate-polyacrylamide gel electrophoresis (SDS-PAGE), followed by electrotransfer onto polyvinylidene fluoride (PVDF) membranes (#IPVH00010, Millipore Sigma, Burlington, VT, USA). The membranes were blocked with 5% non-fat milk dissolved in phosphate-buffered saline (PBS) for 1 h at room temperature, then incubated with corresponding primary antibodies at 4 °C overnight. After washing, the membranes were incubated with matching horseradish peroxidase (HRP)-conjugated secondary antibodies. Protein bands were visualized with an enhanced chemiluminescence (ECL) kit (#BMU101-CN, Abbkine Scientific, Wuhan, China), and signal detection was performed using an ImageQuant 800 imaging station (#ImageQuant 800, Cytiva, Marlborough, MA, USA). The gray intensity of target bands was quantified with ImageJ software (1.53 k).

The primary antibodies used in this study were as follows: anti-p21 (#2947s, Cell Signaling Technology, Danvers, MA, USA); anti-p16 (#A0262, Abclonal, Wuhan, China); anti-β-actin (#ab179467, Abcam, Cambridge, UK); anti-SARM1 (#ab309195, Abcam); anti-cGAS (#A8335, Abclonal); anti-STING (#A3575, Abclonal); anti-TBK1 (#A3458, Abclonal); anti-phospho-TBK1 (#AP1418, Abclonal); anti-IRF3 (#66670-1-Ig, Proteintech, Wuhan, China); anti-phospho-IRF3 (#AP1412, Abclonal). HRP-conjugated goat anti-rabbit IgG (#WLA023, Wanleibio, Shenyang, China) and HRP-conjugated rabbit anti-mouse IgG (#WLA024, Wanleibio) were used as secondary antibodies.

### 2.7. ELISA

The levels of target inflammatory cytokines were detected via enzyme-linked immunosorbent assay (ELISA), with all operations performed strictly in accordance with the manufacturer’s instructions. All ELISA kits used in this study were obtained from Hangzhou Lianke Biotechnology Co., Ltd. (Hangzhou, China), and the detailed kit information with corresponding catalog numbers is listed as follows: Human IL-6 ELISA Kit (#EK106); Mouse IL-6 ELISA Kit (#EK206); Human IL-1β ELISA Kit (#EK101B); Mouse IL-1β ELISA Kit (#EK201B); Human TNF-α ELISA Kit (#EK182); Mouse TNF-α ELISA Kit (#EK282).

### 2.8. Quantification of Cytosolic Mitochondrial DNA (mtDNA)

For the detection of cytosolic mtDNA, cell lysates from HK-2 cells and homogenates of harvested renal tissues were equally divided into two aliquots. One aliquot was digested with Proteinase K (#HY-108717, MedChemExpress) in lysis buffer, followed by purification of total DNA. The remaining aliquot was resuspended in lysis buffer supplemented with Digitonin (#E1321, Selleck Chemicals, Houston, TX, USA) and incubated at room temperature. Sequential centrifugation procedures were performed at 200× *g* for 5 min (repeated twice) and 16,000× *g* for 25 min at 4 °C, to obtain the cytosolic fraction free of nuclear components and intact mitochondria. mtDNA content in the purified cytosolic fraction was quantified via qPCR, with corresponding primer sequences listed in Supplementary Table S1, and the relative mtDNA level was normalized to the total DNA content of the sample.

### 2.9. Quantitative Real-Time PCR (qPCR)

Total RNA was isolated from harvested renal tissues and HK-2 cells using TRIzol reagent (#15596018CN, Invitrogen, Carlsbad, CA, USA), strictly following the manufacturer’s standard operating protocol. The concentration and purity of the extracted RNA were detected using a Nanodrop 2000 spectrophotometer (#Nanodrop 2000, Thermo Fisher Scientific). For complementary DNA (cDNA) synthesis, 2 µg of qualified total RNA was first treated with gDNA Eraser to eliminate genomic DNA contamination, followed by reverse transcription using the PrimeScript RT Reagent Kit (#RR047B, Takara Bio Inc., Kusatsu, Japan). Subsequent qPCR amplification was conducted using SYBR Green Master Mix (#11202ES08, Yeasen Biotechnology, Shanghai, China) on a real-time PCR detection system. The relative expression level of target genes was calculated via the 2^−ΔΔCt^ method, with β-actin serving as the endogenous reference gene for normalization. All primer sequences used for qPCR amplification are listed in Supplementary Table S1.

### 2.10. Transcriptomics Analysis

Transcriptomic sequencing and subsequent bioinformatics analysis were commissioned and completed by Shanghai Personal Biotechnology Co., Ltd. (Shanghai, China). Differential gene expression profiling was performed using R software (version 4.3.3). Genes were identified as significantly differentially expressed genes (DEGs) when meeting the established screening thresholds: |Log_2_(FoldChange)| > 1 and *p*-value < 0.05.

### 2.11. NAD^+^/NADH Assay

The total content of nicotinamide adenine dinucleotide (NAD^+^) and its reduced form (NADH) was quantified via the NAD^+^/NADH Assay Kit (#S0175, Beyotime), strictly following the manufacturer’s standard operating protocol. Briefly, collected samples were incubated with alcohol dehydrogenase working solution and chromogenic reagent at 37 °C under light-proof conditions, with absorbance values subsequently detected at 450 nm. For the independent quantification of NADH alone, an identical aliquot of sample was preheated at 60 °C for 30 min to fully decompose NAD^+^, before being subjected to the same colorimetric reaction procedure. The absolute NAD^+^ content and corresponding NAD^+^/NADH ratio for each sample were calculated based on the detected absorbance data.

### 2.12. Reactive Oxygen Species (ROS) Detection

For renal tissue detection, frozen tissue sections were equilibrated to room temperature, fixed, and subjected to sequential staining with DAPI (#C0060, Solarbio, Beijing, China) and DHE working solution (#D7008-10, Sigma-Aldrich, St. Louis, MO, USA) under light-proof conditions. After sufficient washing with TBST, the sections were mounted with anti-fade mounting medium (#0100-01, Southern Biotech, Birmingham, AL, USA), then stored at 4 °C in a dark environment, and fluorescence imaging was performed using a BX-53 fluorescence microscope (#BX-53, Olympus Corporation, Tokyo, Japan). For HK-2 cells, intracellular ROS levels were quantified using the Reactive Oxygen Species Assay Kit (#S0034S, Beyotime), strictly following the manufacturer’s operating instructions.

### 2.13. Mitochondrial Membrane Potential (ΔΨm) Assay

Mitochondrial membrane potential (ΔΨm) was assessed using the JC-1 fluorescent probe (#C2006, Beyotime). Adherent HK-2 cells were incubated with JC-1 staining working solution at 37 °C for 20 min, washed twice with pre-chilled JC-1 staining buffer, and visualized under a BX-53 fluorescence microscope (Olympus Corporation, Tokyo, Japan). The fluorescence intensities of JC-1 monomers (green fluorescence, Ex/Em ≈ 490/530 nm) and aggregates (red fluorescence, Ex/Em ≈ 525/590 nm) were quantified to reflect relative ΔΨm levels.

### 2.14. Transmission Electron Microscopy (TEM)

Fresh renal tissues were rapidly trimmed into 1 mm^3^ cubes, and HK-2 cells were scraped and centrifuged to form 1–2 mm^3^ pellets. All samples were pre-fixed with 2.5% glutaraldehyde, post-fixed with 1% osmium tetroxide, dehydrated through graded ethanol and acetone, infiltrated and embedded in 812 resin, then polymerized at 60 °C. 70 nm ultrathin sections were cut with an ultramicrotome, collected on copper grids, stained with uranyl acetate and lead citrate, and observed under a transmission electron microscope (all instruments and materials for this process were provided by Hubei BIOSSCI Biotechnology Co., Ltd.).

### 2.15. Molecular Docking and Molecular Dynamics (MD) Simulation

The 3D structure of DP was retrieved from PubChem (https://pubchem.ncbi.nlm.nih.gov/) (accessed on 15 September 2025) and converted to PDB format via PyMol (3.1.0). The crystal structure of target protein SARM1 (PDB ID: 7CM5) was obtained from RCSB PDB (https://www.rcsb.org/) (accessed on 15 September 2025), with intrinsic ligands removed using PyMol. Both DP and protein were pretreated by dehydration, hydrogenation and charge calculation with AutoDockTools (4.2.6), then saved as PDBQT files. Molecular docking was performed using AutoDock Vina, and results were visualized in PyMol.

MD simulations were conducted using GROMACS. DP and SARM1 structures were parameterized with the AMBER ff14SB force field, solvated in a TIP3P water box, and neutralized with 150 mM NaCl. After energy minimization, the system was equilibrated under NVT and NPT ensembles for 100 fs each, followed by a 100-ns production run with a 10-fs time step. Trajectory data were saved every 10 fs for RMSD and Rg analysis to assess system stability.

### 2.16. Surface Plasmon Resonance (SPR)

Surface plasmon resonance (SPR) analysis was performed by Beijing Bioteke Co., Ltd. (Beijing, China). Following the manufacturer’s standard guidelines, a three-step procedure was used to determine the binding affinity of DP to SARM1 protein, including chip preparation, ligand immobilization, and multi-cycle kinetic analysis.

### 2.17. Immunofluorescence and Immunohistochemistry (IHC) Staining

For multiplex immunofluorescence staining, the TSA method was adopted. Paraffin sections were deparaffinized, antigen-retrieved, and blocked for endogenous peroxidase, then subjected to three sequential rounds of antigen labeling. Each round included incubation with primary antibody (4 °C, overnight), secondary antibody (37 °C, 45 min), and tyramide working solution (room temperature, 10 min, light-proof). After each round, antibody complexes were gently eluted (42 °C, 20 min) for subsequent labeling. Finally, nuclei were counterstained with DAPI (#C0060, Solarbio), sections mounted with anti-fade mounting medium (#0100-01, Southern Biotech), stored at 4 °C in the dark, and imaged via a BX-53 fluorescence microscope.

For IHC staining, post serum blocking, sections were incubated with primary antibodies (4 °C, overnight) and corresponding secondary antibodies (37 °C, 45 min). DAB color development was performed, followed by hematoxylin counterstaining, dehydration, clearing and neutral resin mounting. Images were acquired with a light microscope.

The primary antibodies used were: anti-SARM1 (#ab309195, Abcam); anti-NDUFB8 (#T58290S, Abmart, Shanghai, China); anti-TOMM20 (#ab56783, Abcam).

### 2.18. Statistical Analysis

All statistical analyses were performed using GraphPad Prism software (version 7.00). Data are presented as mean ± standard deviation. The specific sample size (*n*) and number of independent replicates are indicated in the corresponding figure legends. The Kolmogorov–Smirnov test was used to assess normality. Comparisons between two groups were analyzed using Student’s *t*-test. Comparisons among multiple groups were analyzed by one-way or two-way analysis of variance (ANOVA), followed by Bonferroni’s post hoc test. A *p*-value < 0.05 was considered statistically significant.

## 3. Results

### 3.1. DP Alleviates DG-Induced HK-2 Cell Senescence by Suppressing SASP

**Figure 1:**
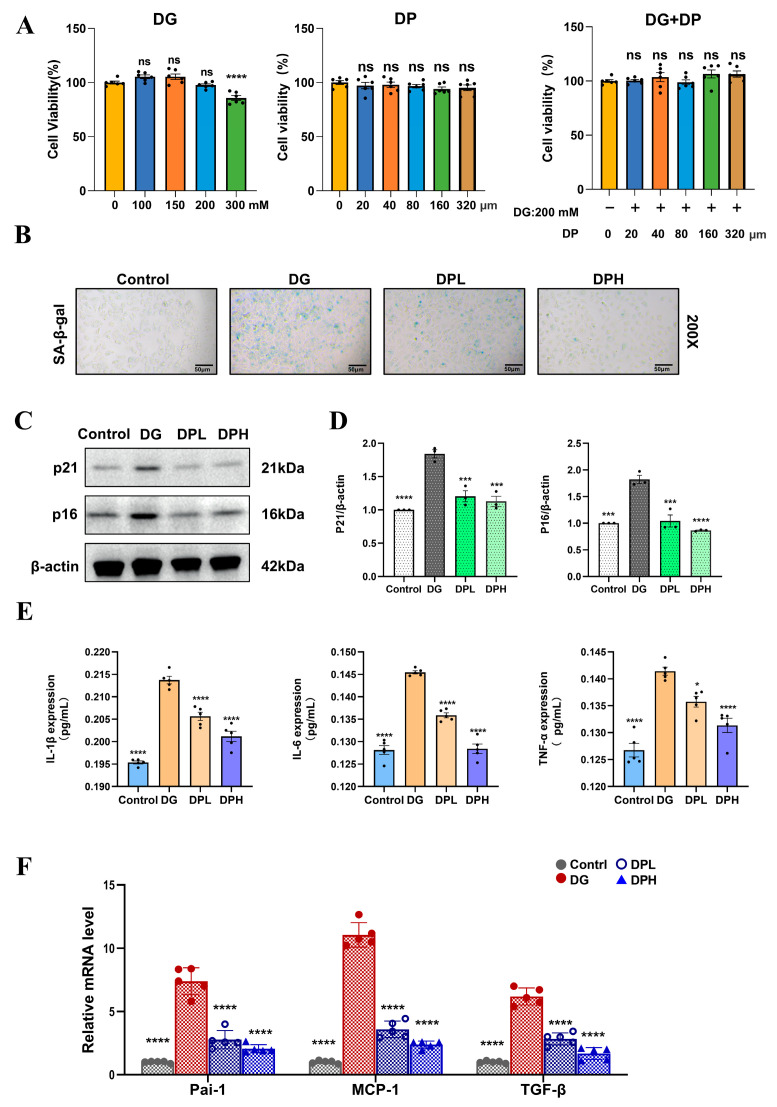
DP alleviates DG-induced HK-2 cell senescence by suppressing SASP. (**A**) CCK-8 assay evaluating the effects of DG, DP, and their combination on HK-2 cell viability. The concentration of 200 mM DG was selected because it effectively induced senescence markers (SA-β-gal positivity, p21/p16 expression) without excessive cytotoxicity. (**B**) SA-β-gal staining of HK-2. Scale bar: 50 μm. (**C**,**D**) Western blot analysis of p21 and p16 protein levels. (**E**) ELISA measuring the expression levels of IL-1β, IL-6, and TNF-α in cell culture supernatants. (**F**) qPCR detection of Pai-1, MCP-1, and TGF-β mRNA expression in HK-2. Data are expressed as mean ± SD. Statistical significance is indicated as * *p* < 0.05, *** *p* < 0.001; **** *p* < 0.0001; ns, not significant.

To determine the experimental conditions for inducing senescence in HK-2 cells, the cells were treated with DP, DG, or a combination of both for 24 h. Based on cell viability assessed by the CCK-8 assay (Figure 1A), the final intervention concentrations were set at 200 mM for DG, and 160 µM (low dose, DPL) or 320 µM (high dose, DPH) for DP. As shown in Figure 1B, DP treatment reduced the elevated SA-β-gal activity induced by DG. On one hand, DP decreased the protein expression of senescence markers p21 and p16 in HK-2 cells (Figure 1C,D). On the other hand, ELISA of cell culture supernatants (Figure 1E) demonstrated that DP effectively reduced the levels of senescence-associated inflammatory cytokines (IL-1β, IL-6, TNF-α). Concurrently, the mRNA levels of other SASP factors (Pai-1, MCP-1, TGF-β) were also ameliorated (Figure 1F). These results lead to the preliminary conclusion that DP delays renal aging by inhibiting SASP release and alleviating inflammation.

### 3.2. Transcriptomics Reveals SARM1 as a Potential Target for the Anti-Aging Effect of DP

**Figure 2:**
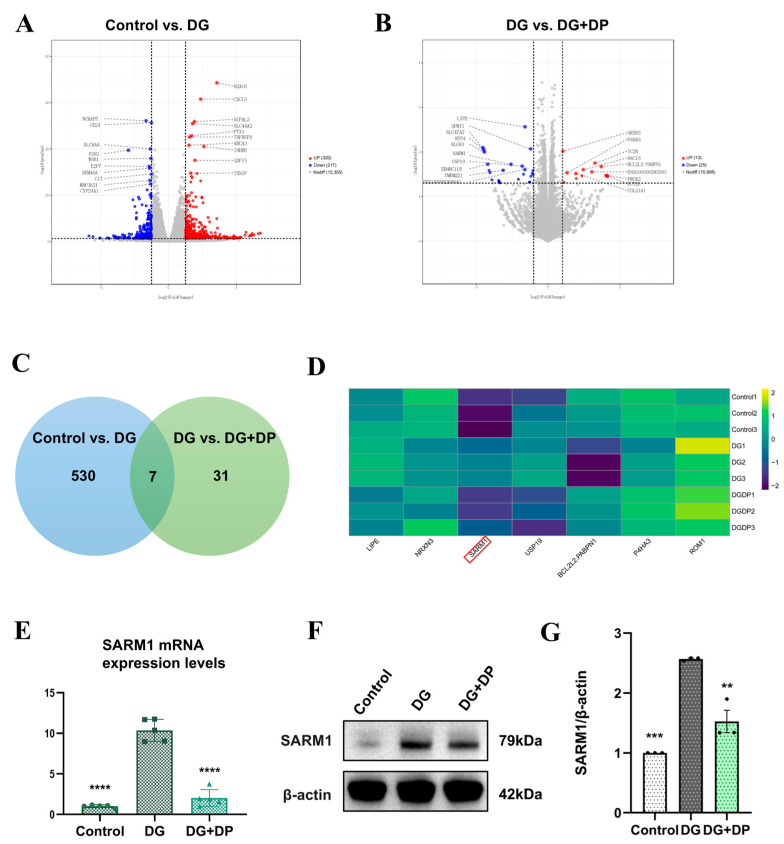
Transcriptomics reveals SARM1 as a potential target for the anti-aging effect of DP. (**A**,**B**) Volcano plots showing differentially expressed genes. (**C**) Venn diagram displaying the overlapping differentially expressed genes. (**D**) Heatmap illustrating the expression patterns of the overlapping genes across groups. Red box: SARM1. (**E**) qPCR analysis showing the mRNA level of SARM1 in HK-2. (**F**,**G**) Western blot analysis detecting SARM1 protein levels in HK-2. Data are expressed as mean ± SD. Statistical significance is indicated as ** *p* < 0.01, *** *p* < 0.001; **** *p* < 0.0001.

Having established that DP delays DG-induced senescence in HK-2 cells, we performed transcriptomic analysis on the Control, DG, and DG + DP (320 µM DP) groups to further investigate the mechanism. Differential expression analysis revealed that DG intervention upregulated 320 genes and downregulated 217 genes. Compared to the DG group, DP intervention led to the upregulation of 13 genes and downregulation of 25 genes (Figure 2A,B). A Venn diagram identified 7 genes differentially expressed across all three groups (Figure 2C), and their expression patterns are presented in a heatmap (Figure 2D). Among them, the differential expression of SARM1 attracted our interest, as its activation consumes NAD^+^ and leads to mitochondrial dysfunction—a key hallmark of aging. Subsequent qPCR detection of SARM1 expression in HK-2 cells validated the transcriptomic sequencing results (Figure 2E). Notably, SARM1 protein expression showed a trend consistent with its mRNA expression (Figure 2F,G).

### 3.3. Inhibition of SARM1 Alleviates Renal Aging by Improving Mitochondrial Function

**Figure 3:**
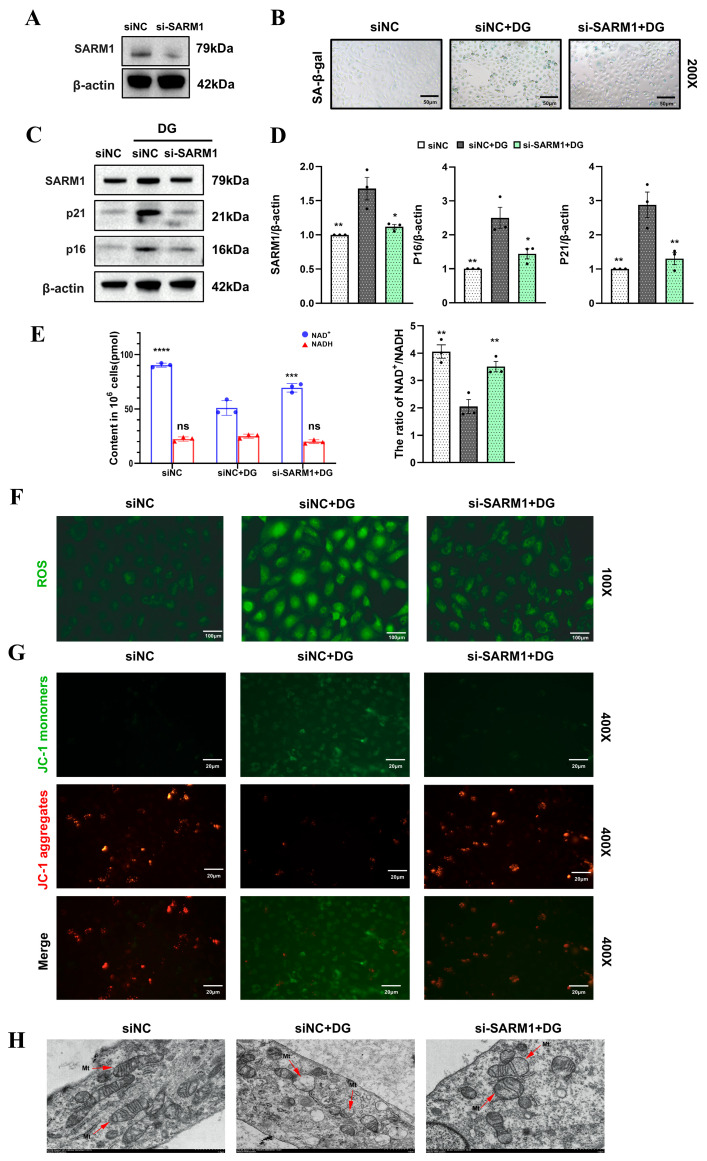
Inhibition of SARM1 alleviates renal aging by improving mitochondrial function. (**A**) SARM1 protein expression after siRNA intervention. (**B**) SA-β-gal staining of HK-2. Scale bar: 50 μm. (**C**,**D**) Protein levels of p21 and p16 in HK-2 following SARM1 siRNA transfection. (**E**) Content and ratio of NAD^+^ and NADH in HK-2. (**F**) ROS expression (green fluorescence) in HK-2. Scale bar: 100 μm. (**G**) JC-1 staining for detection of mitochondrial membrane potential. Prominent JC-1 aggregates (red) indicate higher mitochondrial membrane potential, whereas predominant JC-1 monomers (green) reflect lower potential. Scale bar: 20 μm. (**H**) Representative transmission electron microscopy images of HK-2 in each group. Red arrows indicate mitochondria. Scale bar: 1 μm. Data are expressed as mean ± SD. Statistical significance is indicated as * *p* < 0.05, ** *p* < 0.01, *** *p* < 0.001; **** *p* < 0.0001, ns, not significant.

Given the lack of studies on the role of SARM1 in renal aging, we used siRNA to silence SARM1 expression followed by DG intervention to explore its significance in HK-2 cell senescence. SARM1 protein levels decreased after siRNA intervention (Figure 3A). Subsequent experiments showed that inhibition of SARM1 expression effectively delayed senescence progression, as indicated by reduced intracellular SA-β-gal activity (Figure 3B) and decreased expression of senescence-related proteins p21 and p16 (Figure 3C,D). Since SARM1 activation consumes large amounts of NAD^+^, we observed that the NAD^+^ level and NAD^+^/NADH ratio in the SARM1 inhibition group did not decline rapidly as in the DG group (Figure 3E). Further experiments indicated that inhibiting SARM1 expression reduced the DG-elevated intracellular ROS levels (Figure 3F) and helped maintain mitochondrial membrane potential at a relatively favorable level (Figure 3G). Transmission electron microscopy (TEM) provided a direct visualization of mitochondrial status across groups: DG intervention caused significant mitochondrial swelling, cristae rupture, and matrix vacuolization (Figure 3H), whereas mitochondrial morphology in the SARM1 inhibition group was only mildly affected. These results suggest that the delay in renal aging by SARM1 inhibition is closely associated with improved mitochondrial function.

### 3.4. DP Stably Binds to SARM1

**Figure 4:**
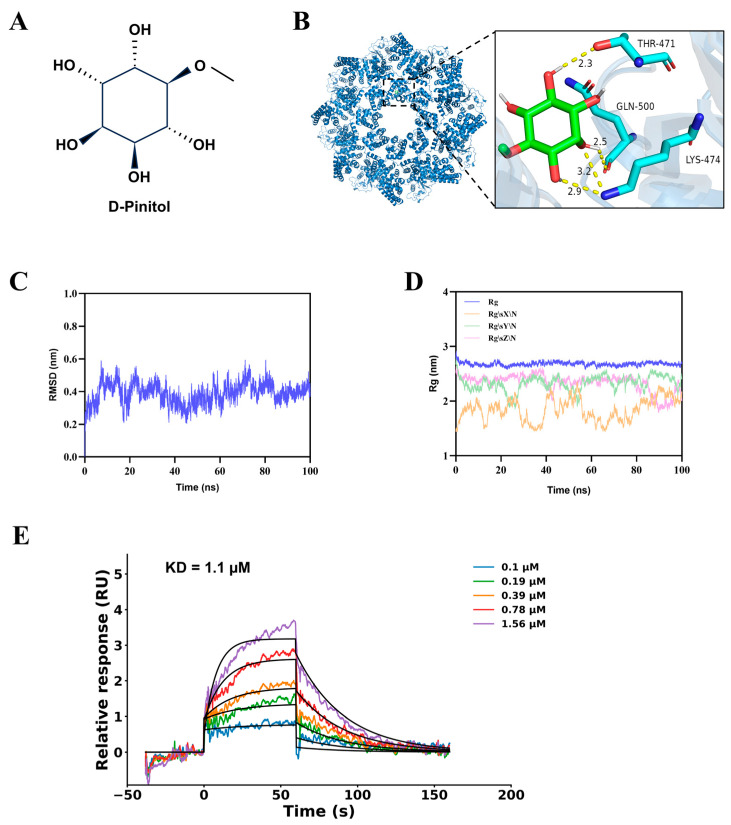
DP stably binds to SARM1. (**A**) Chemical structure of D-pinitol (DP). (**B**) Molecular docking model illustrating the hydrogen-bond interactions between DP and the SARM1 protein (PDB ID: 7CM5). (**C**,**D**) Indicators from molecular dynamics simulations. (**E**) Surface plasmon resonance results for the binding between DP and the SARM1 protein.

To investigate whether there is a direct interaction between DP (Figure 4A) and the SARM1 protein, we first performed molecular docking simulations. These simulations yielded a predicted binding free energy of −5.6 kcal/mol for the DP-SARM1 complex. Detailed structural dissection of the docking results revealed that multiple hydroxyl groups on the DP molecule form stable hydrogen bond interactions with three amino acid residues of SARM1: THR471 (bond length 2.3 Å), GLN500 (bond length 2.5 Å), and LYS474 (bond lengths 2.9 Å and 3.2 Å) (Figure 4B). This binding pattern suggests that DP may directly target the SAM domain of the SARM1 protein. We then carried out molecular dynamics simulations to verify the stability of this predicted binding conformation. Root mean square deviation (RMSD) analysis of the simulation trajectories indicated high structural stability of the DP-SARM1 complex system, which only showed mild fluctuations throughout the simulation process, reached a stable equilibrium state after 85 ns, and maintained an overall fluctuation amplitude of approximately 0.4 nm. Additional radius of gyration (Rg) analysis further supported that the complex system maintained a tightly packed, compact structural conformation during the simulation (Figure 4C,D). At the protein level, we used surface plasmon resonance (SPR) assays to experimentally examine the direct binding between DP and SARM1. The assay results provided evidence for a specific interaction between DP and the SARM1 protein, with an equilibrium dissociation constant (K_D) of 1.1 µM (Figure 4E).

### 3.5. DP Inhibits SARM1 to Reduce mtDNA Leakage, Thereby Suppressing the cGAS-STING Signaling Pathway

**Figure 5:**
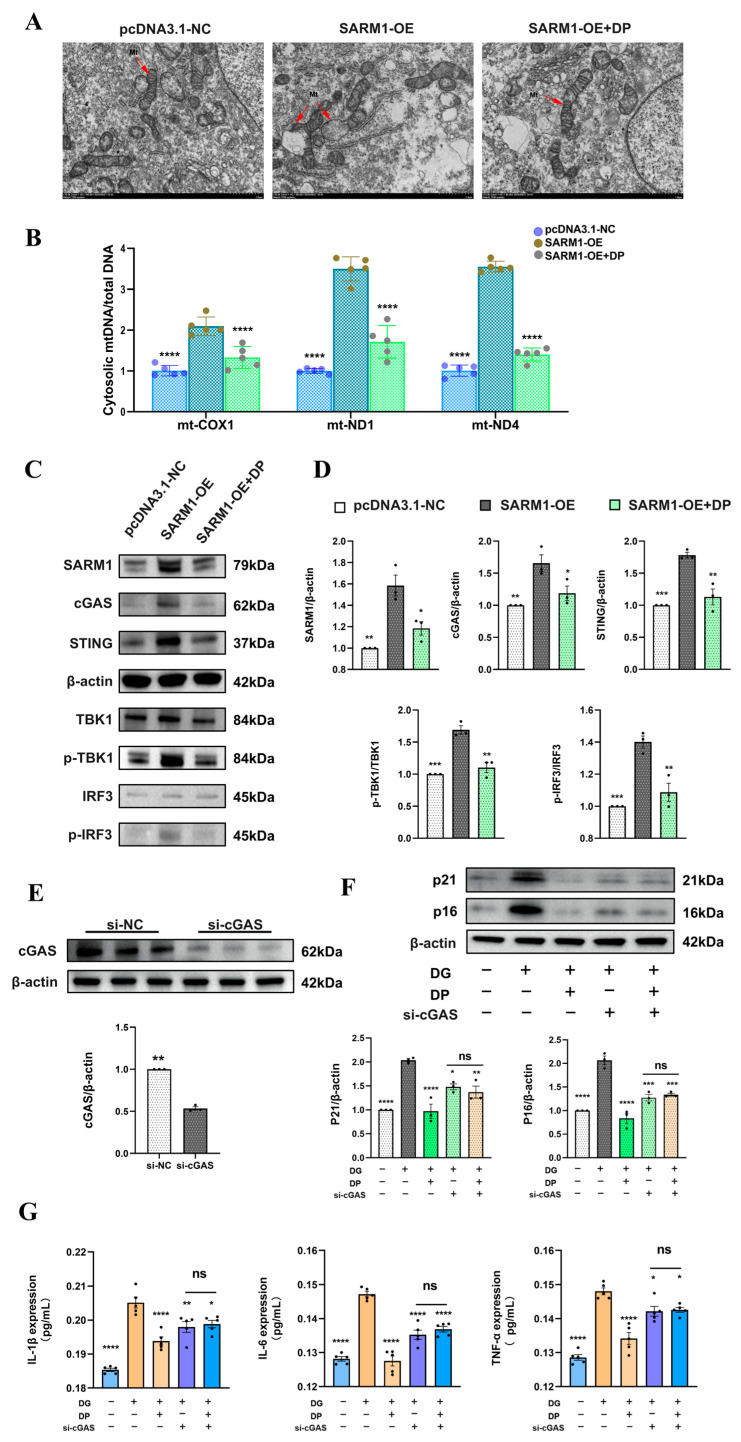
DP inhibits SARM1 to reduce mtDNA leakage, thereby suppressing the cGAS-STING signaling pathway. (**A**) Transmission electron microscopy results. Scale bar: 1 μm. (**B**) Quantitative PCR analysis of mitochondrial DNA (mtDNA), including COX 1, ND1, and ND4, in HK 2. (**C**,**D**) Expression levels and quantitative analysis of proteins related to the cGAS–STING pathway. (**E**) cGAS protein expression after siRNA intervention. (**F**) Protein expression levels of p21 and p16. (**G**) ELISA measuring the expression levels of IL-1β, IL-6, and TNF-α in cell culture supernatants. Data are expressed as mean ± SD. Statistical significance is indicated as * *p* < 0.05, ** *p* < 0.01, *** *p* < 0.001; **** *p* < 0.0001, ns, not significant.

With the confirmed regulatory effect of DP on SARM1 inhibition and mitochondrial function preservation, we further explored the mechanistic link between this regulatory action and the suppression of SASP factor generation. We generated SARM1-overexpressing HK-2 cells via transfection with the pcDNA3.1-SARM1 recombinant plasmid, then performed transmission electron microscopy (TEM) to observe mitochondrial morphology in these cells. TEM imaging results showed severe impairment of mitochondrial structure, including obvious rupture of the mitochondrial membrane, in cells with SARM1 overexpression (Figure 5A). Based on these morphological changes, we proposed a potential mechanistic cascade: mitochondrial structural damage triggers the release of mitochondrial DNA (mtDNA) into the cytoplasm, where the ectopic mtDNA is recognized by cGAS, which in turn activates its downstream signaling cascade and drives the generation of SASP factors. Subsequent experimental validation supported this hypothesis, as we detected a marked elevation in the cytosolic levels of three representative mtDNA markers, namely COX-1, ND1 and ND4, in the SARM1-overexpressing cell group (Figure 5B). In parallel with the mtDNA leakage, we also found a significant upregulation in the protein expression levels of core molecules in the cGAS-STING innate immune signaling pathway (Figure 5C,D). In line with the rescue effect of DP on mitochondrial structural damage, all the above-mentioned abnormal upregulations induced by SARM1 overexpression were effectively reversed after DP intervention. To further verify whether the cGAS-STING pathway is essential for the anti-senescence effect of D-pinitol, we silenced cGAS expression in HK-2 cells using siRNA (Figure 5E). Under DG-induced stress, cGAS silencing alone effectively decreased the protein levels of p21 and p16, as well as the levels of IL-1β, IL-6, and TNF-α in the cell culture supernatant. (Figure 5F,G). These findings are consistent with the known role of the cGAS-STING pathway. Notably, additional DP treatment in cGAS-silenced cells failed to induce further reduction in these senescence markers and cytokines. These findings suggest that the cGAS-STING pathway is a potential key mediator of the anti-aging effect of DP.

### 3.6. DP Ameliorates DG-Induced Renal Aging in Mice

**Figure 6:**
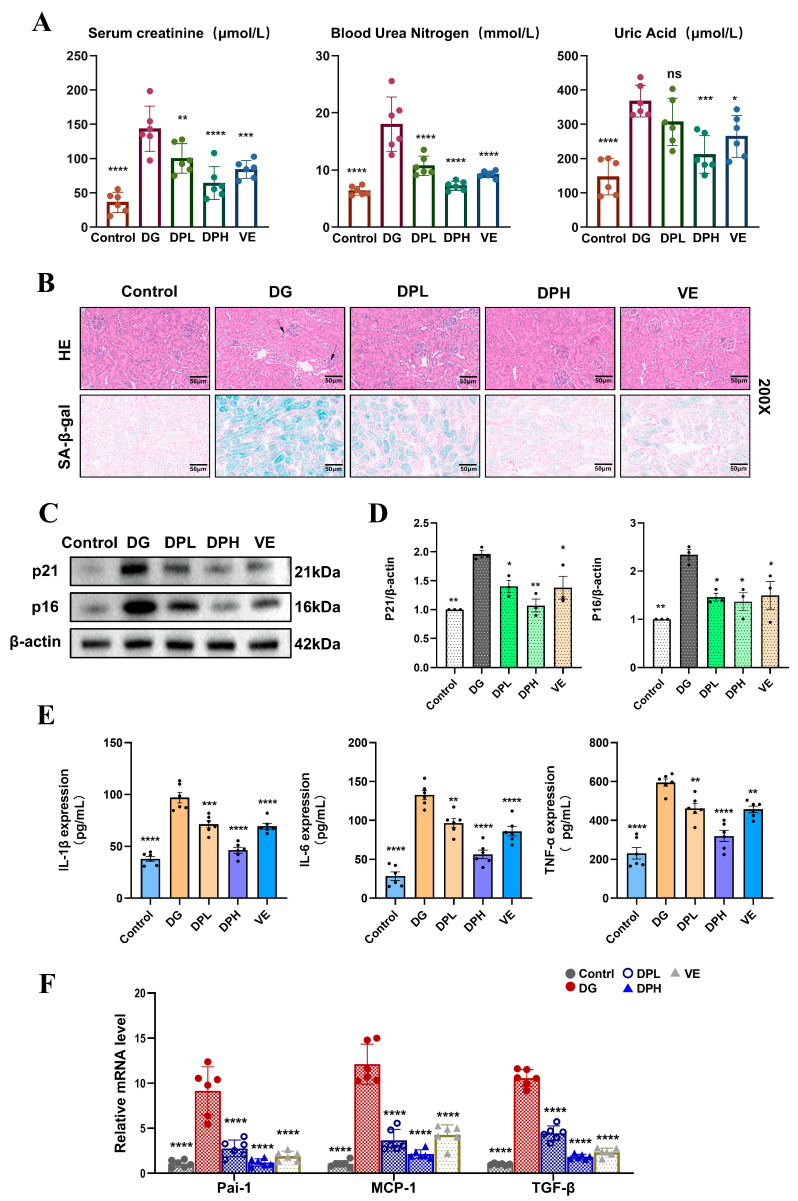
DP ameliorates DG-induced renal aging in mice. (**A**) Levels of serum uric acid, creatinine, and blood urea nitrogen in male C57BL/6J mice from each group. (**B**) Representative images of HE staining and SA-β-gal staining in mouse kidney sections. The arrows point to the sites of injury. Scale bar: 50 μm. (**C**,**D**) Expression and quantitative analysis of senescence-associated proteins p21 and p16 in mouse kidney tissues. (**E**) Expression levels of IL-6, IL-1β, and TNF-α in mouse serum were detected by ELISA. (**F**) qPCR detection of Pai-1, MCP-1, and TGF-β mRNA expression. Data are expressed as mean ± SD. Statistical significance is indicated as * *p* < 0.05, ** *p* < 0.01, *** *p* < 0.001; **** *p* < 0.0001; ns, not significant.

Building on the anti-senescence and anti-inflammatory effects of DP observed in cellular models, we verified its renal protective efficacy against aging-related injury in C57BL/6J mouse models in vivo. We established a systemic renal aging mouse model via 6-week continuous subcutaneous administration of D-galactose (DG) at a daily dose of 250 mg/kg body weight. During the entire modeling period, mice received daily oral gavage of DP at two dose gradients: 50 mg/kg/d for the low-dose group (DPL) and 100 mg/kg/d for the high-dose group (DPH), with 100 mg/kg/d vitamin E supplementation set as the positive control group. Functional assessment results first demonstrated that DP intervention effectively alleviated DG-induced renal function impairment in mice (Figure 6A). Histological staining of renal tissues further revealed that the DG model group presented severe renal structural lesions, including marked inflammatory cell infiltration, swelling and structural deformation of tubular epithelial cells, along with obvious vacuolar degeneration. SA-β-gal staining of the same tissue sections also showed a significant increase in senescence-positive cell proportion in the DG group (Figure 6B). DP treatment notably ameliorated these pathological structural damages in renal tissues, and significantly downregulated SA-β-gal activity, with a rescue effect comparable to that of the vitamin E positive control group. In line with the histological findings, Western blot assays confirmed that DP intervention effectively reversed the DG-induced abnormal upregulation of the core senescence-related proteins p21 and p16 in mouse renal tissues (Figure 6C,D). We then detected circulating inflammatory factors in mouse serum via ELISA, and found that DG exposure triggered a marked elevation in the levels of pro-inflammatory cytokines including IL-1β, IL-6 and TNF-α (Figure 6E), which was effectively reversed by DP administration. Consistent with the changes in circulating inflammatory profiles, qPCR detection of mouse renal tissue samples showed that the mRNA expression levels of additional SASP-related factors, including Pai-1, MCP-1 and TGF-β, were significantly increased in the DG model group, and this abnormal upregulation was also markedly ameliorated by DP intervention (Figure 6F).

### 3.7. SARM1 Mediates the Improvement of Renal Mitochondrial Dysfunction by DP in Aging Mice

**Figure 7:**
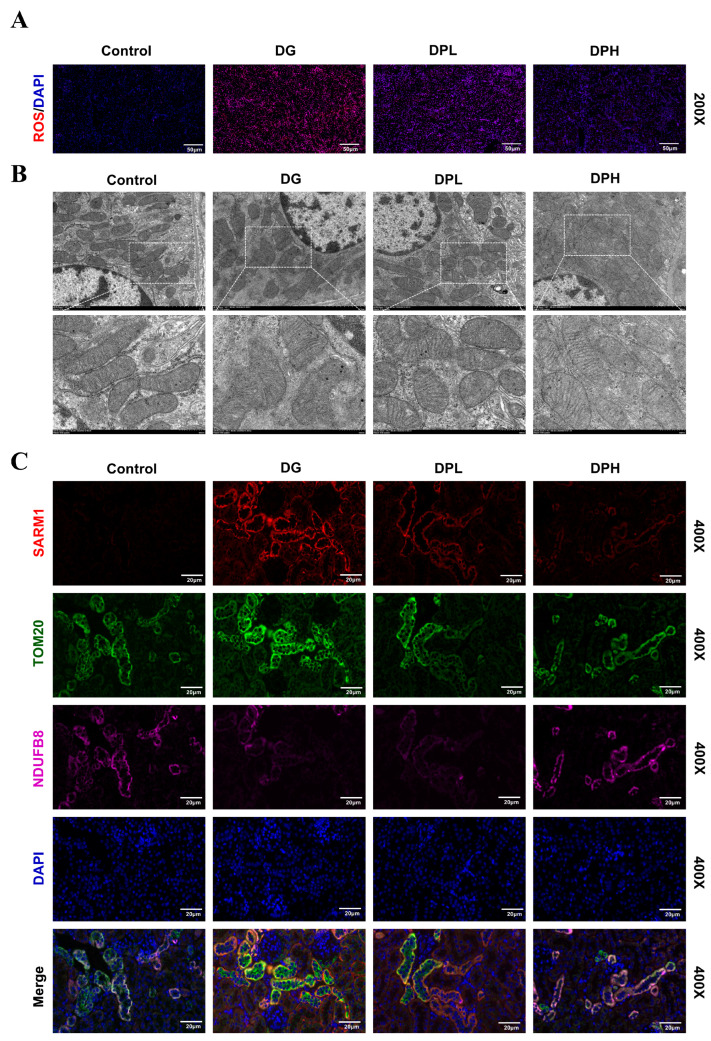
SARM1 mediates the improvement of renal mitochondrial dysfunction by DP in aging mice. (**A**) ROS levels in mouse kidney tissues (red fluorescence). Scale bar: 50 µm. (**B**) Transmission electron microscopy images of the mouse renal cortex. Scale bar: 2 µm and 500 nm. (**C**) Representative immunofluorescence staining images of mouse kidney tissues showing SARM1 (red), TOM20 (green), NDUFB8 (purple), and DAPI (blue). Scale bar: 20 µm.

To further explore the intrinsic mechanism of DP’s renal protective effect in vivo, we first detected reactive oxygen species (ROS) accumulation in mouse renal tissues. Fluorescence detection results presented in Figure 7A showed that DG exposure induced a prominent increase in ROS fluorescence intensity in mouse renal tissues, whereas DP administration, particularly in the high-dose DPH group, effectively suppressed this abnormal ROS overproduction. Given the close association between excessive ROS generation and mitochondrial structural damage, we then used transmission electron microscopy (TEM) to observe ultrastructural changes in renal cortical cells across all experimental groups (Figure 7B). Renal tubular epithelial cells from DG-treated mice exhibited severe mitochondrial morphological lesions, including marked swelling, extensive fragmentation of mitochondrial cristae, and even complete cristae disappearance. By contrast, DP intervention effectively maintained the normal structural morphology of mitochondria in renal tubular epithelial cells, with a notably improved mitochondrial ultrastructure compared with the DG model group. To verify whether the mitochondrial protective effect of DP in vivo is mediated by SARM1 inhibition, we performed triple immunofluorescence staining on renal tissue sections. Staining results displayed in Figure 7C revealed that the protein expression of SARM1 (red fluorescence) was markedly upregulated in renal tissues of the DG model group, accompanied by a significantly increased co-localization level between SARM1 and the mitochondrial marker TOM20 (green fluorescence). More notably, the fluorescence signal of NDUFB8 (purple fluorescence), a core functional subunit of mitochondrial respiratory chain complex I, was significantly reduced in renal tissues of the DG group. DP treatment effectively reversed the abnormal upregulation of SARM1, decreased its co-localization with TOM20, and simultaneously markedly restored both the fluorescence signal intensity and mitochondrial localization pattern of NDUFB8 in renal tissues.

### 3.8. The SARM1-cGAS-STING Axis Is Involved in the Anti-Aging Effects of DP In Vivo

**Figure 8:**
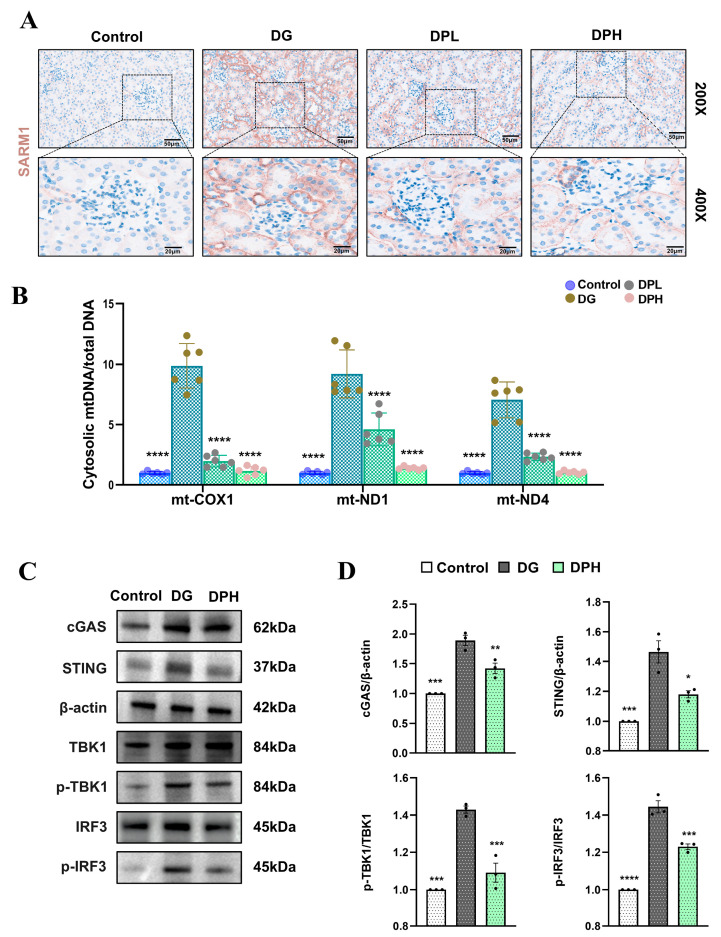
The SARM1-cGAS-STING axis is involved in the anti-aging effects of DP in vivo. (**A**) Immunohistochemical staining of mouse kidney sections showing the localization and expression level of SARM1. Scale bar: 50 µm and 20 µm. (**B**) qPCR detection of mitochondrial markers (COX 1, ND1, ND4) in kidney tissues. (**C**,**D**) Expression levels of proteins related to the cGAS–STING signaling pathway in kidney tissues. Data are expressed as mean ± SD. Statistical significance is indicated as * *p* < 0.05, ** *p* < 0.01, *** *p* < 0.001; **** *p* < 0.0001.

Immunohistochemical staining results showed that SARM1 protein expression was extremely low in the kidney tissues of control mice (Figure 8A). In the DG group, SARM1 expression was significantly upregulated. Interestingly, this upregulation specifically enriched in renal tubules, while glomeruli maintained very low levels. Consistent with in vitro results, the upregulation of SARM1 was effectively reversed by DP. Furthermore, we measured cytosolic mtDNA markers (Figure 8B) and the expression levels of cGAS-STING pathway proteins (Figure 8C,D). These results validated our in vitro conclusions: DP inhibits SARM1, reduces mtDNA leakage, and thereby suppresses the cGAS-STING signaling pathway.

## 4. Discussion

The crucial role of SARM1 (Sterile Alpha and TIR Motif Containing 1) in axon degeneration and neurodegenerative diseases has been extensively documented [17]. Recent research has broadened the scope of this target. For instance, one study proposed SARM1 as a novel anti-fibrotic target in the heart, where its upregulation post-myocardial infarction, stabilized by palmitoylation, directly regulates P4HA1 to promote cardiac fibrosis [18]. In liver fibrosis research, SARM1 forms a functional coupling with TRPV1, recruited by the latter to maintain hepatic stellate cell quiescence [19]. SARM1 contains three domains from the N- to C-terminus: ARM, SAM, and TIR. At rest, the ARM domain interacts with the TIR domain, effectively “locking” it and preventing its NAD^+^ hydrolase (NADase) activity [20]. Under stress conditions like oxidative stress, the SAM domain senses metabolic stress signals and initiates oligomerization. The resulting allosteric tension is transmitted to the ARM domain, promoting ARM-TIR dissociation. The released TIR domains then form a stable dimeric catalytic interface within the SAM-mediated oligomeric framework, thereby acquiring potent NAD^+^ hydrolase activity and leading to rapid intracellular NAD^+^ depletion [21]. NAD^+^ is critically important for mitochondrial energy metabolism and functional regulation [22]. In our aging model, we observed abnormal activation of SARM1 and a consequent significant decrease in NAD^+^, which was closely associated with mitochondrial dysfunction and structural damage. A recent study in acute kidney injury showed that METTL1-mediated post-transcriptional modification of SARM1 mRNA promotes SARM1 protein expression, leading to NAD^+^ depletion and metabolic reprogramming in macrophages, ultimately exacerbating inflammation [23]. Our investigation into the role of SARM1 in renal aging revealed its tubule-specific expression in the kidneys of DG-induced aging mice. Importantly, both the inhibitory effect of DP and the mitochondrial functional improvement resulting from SARM1 silencing in vitro effectively delayed renal aging. Molecular docking simulations suggested a potential interaction between DP and SARM1, indicating that DP may directly bind to the SAM domain of SARM1. We observed that DP reversed DG-induced upregulation of SARM1 and the associated marked decline in NAD^+^ levels. However, the lack of NADase activity assays in the present study precludes definitive validation of whether DP exerts a functional inhibitory effect on the enzymatic activity of SARM1. Based on the docking results and the observed changes in NAD^+^ levels, we hypothesize that D-pinitol might interfere with the “signal sensor” function of the SAM domain, potentially acting as an allosteric inhibitor. This hypothesis warrants direct testing in future studies using in vitro NADase activity assays. Notably, we also observed that DP downregulated the mRNA and protein levels of SARM1, which may be attributed to a potential negative feedback loop triggered by the inhibition of SARM1 enzymatic activity. This potential regulatory mechanism will also be systematically explored in our follow-up studies.

Mitochondrial dysfunction-associated senescence is considered a type of aging induced by SASP driven through the NADH/AMPK/p53 pathway [24]. Mitochondrial dysfunction is a key driver of cellular senescence. Renal tubular epithelial cells possess a remarkably dense mitochondrial network and rely heavily on mitochondrial oxidative phosphorylation for their energy-demanding functions. Therefore, they are the primary cell type that maintains the kidney’s high metabolic rate [25]. This high workload makes their mitochondria more vulnerable to cumulative oxidative stress damage. Such damage may cause outer mitochondrial membrane rupture, leading to the release of mitochondrial DNA (mtDNA) into the cytosol [26]. Cytosolic mtDNA is specifically recognized by the DNA sensor cyclic GMP-AMP synthase (cGAS), which catalyzes the synthesis of the second messenger cGAMP. cGAMP then activates the adaptor protein STING on the endoplasmic reticulum membrane, triggering the downstream TBK1 kinase and the activation and nuclear translocation of transcription factors IRF3 and NF-κB. This process directly drives the transcription and release of SASP, the core manifestation of inflammaging [27,28]. While several pharmacological interventions targeting SASP (such as metformin, rapamycin, and ruxolitinib) have demonstrated anti-senescence effects in preclinical studies, robust clinical validation remains limited [29]. Our study reveals that DP similarly exerts anti-senescence effects by modulating SASP, and this action is achieved through protecting mitochondrial function in renal tubular epithelial cells.

DP is a potential mediator involved in insulin signaling. Long-term intake has been shown to reduce blood glucose and increase insulin sensitivity in healthy or prediabetic subjects [30], while also improving endothelial dysfunction and oxidative stress in patients with type 2 diabetes [31]. Its clear pharmacological activity and favorable safety profile have been preliminarily confirmed in clinical studies. Pharmacokinetic research in healthy individuals indicates that oral DP is rapidly absorbed into the bloodstream when administered alone (compared to co-administration with carbohydrates), exhibiting a longer absorption period and half-life [32]. Incorporating DP as a dietary component could be a reasonable strategy for addressing insulin resistance or type 2 diabetes in many developed countries. Another clinical study involving an obese population demonstrated that oral DP significantly reduced the expression of inflammatory cytokines (IL-6, TNF-α) in the blood [33]. The hypoglycemic, anti-inflammatory, and antioxidant effects of DP have been repeatedly confirmed. We hope to uncover more of its potential clinical value, providing new avenues for treating chronic metabolic diseases. While one study found that DP ameliorated symptoms in a mouse model of diabetic sarcopenia [34], few studies have systematically discussed the anti-aging mechanism of DP from both in vivo and in vitro perspectives, particularly concerning the kidney. Our study provides evidence supporting that DP delays renal aging via inhibiting SARM1 to protect renal tubular epithelial cells from mitochondrial damage, which has not been systematically reported in previous in vitro and in vivo studies.

Although our experimental results provide meaningful insights, several limitations of this study should be acknowledged. The immortalized nature of HK-2 cells prevents them from fully recapitulating the hallmarks of replicative senescence, and the in vitro monoculture system fails to capture the complex cellular and systemic interactions involved in physiological renal aging. The D-galactose-induced aging model, while widely used, primarily reflects oxidative and metabolic stress and does not completely reproduce the multifactorial complexity of physiological aging. Therefore, future studies using naturally aged animal models and in vivo models for SARM1 gene editing are warranted to validate the conclusion that DP exerts anti-aging effects by inhibiting the SARM1–cGAS–STING axis. Furthermore, given the differential susceptibility of tubule segments to senescence-related injury, future studies should precisely define SARM1 distribution and explore potential segment-specific functional differences.

In summary, this study provides evidence identifying SARM1 as a key target in renal aging and suggests that the food-derived cyclitol D-pinitol counteracts this process, likely via stabilizing the auto-inhibitory conformation of SARM1. By preserving mitochondrial homeostasis and inhibiting the cGAS-STING pathway, D-pinitol offers a novel, mechanism-based strategy to counteract age-related renal impairment, underscoring its potential for nutraceutical applications.

## Data Availability

The original contributions presented in this study are included in the article/Supplementary Materials. Further inquiries can be directed to the corresponding author.

## Supplementary Materials

The following supporting information can be downloaded at: https://www.mdpi.com/article/10.3390/biomedicines14051092/s1, Table S1: Primers used for qPCR analysis.
