# Supplementary material for: D-Pinitol Mitigates Renal Senescence via Targeting the SARM1-cGAS-STING Signaling Axis to Restore Mitochondrial Function and Dampen Inflammatory Responses
Source: Biomedicines. 2026 May 12;14(5):1092. doi: 10.3390/biomedicines14051092 (PMC13204088; doi:10.3390/biomedicines14051092)
Supplement: Supplementary file 1 [file biomedicines-14-01092-s001.zip › biomedicines-4304590-supplementary.pdf]

Table S1: Primers used for qPCR analysis.

Species	Name	Sequences
Mice	mtCOX-1 Forward (5' - 3')	GATTGTACTCGCACGGGCTAC
	mtCOX-1 Reverse (5' - 3')	GGATAAAGGTTGGAVVGCACT
	mtND1 Forward (5' - 3')	TCCGAGCATCTTATCCACGC
	mtND1 Reverse (5' - 3')	GTATGGTGGTACTCCCGCTG
	mtND4 Forward (5' - 3')	ATAATCGCACATGGCCTCA
	mtND4 Reverse (5' - 3')	GGATCCGTTCGTAGTTGG
	Pai-1 Forward (5' - 3')	CTCCACAGCCTTTGTCATCT
	Pai-1 Reverse (5' - 3')	ATTGTCTCTGTCGGGTTGTG
	TGF-β1 Forward (5' - 3')	CGAAGCGGACTACTATGCTAAA
	TGF-β1 Reverse (5' - 3')	TCCCGAATGTCTGACGTATTG
	MCP-1 Forward (5' - 3')	CTTCTGGGCCTGCTGTTCA
	MCP-1 Reverse (5' - 3')	CCAGCCTACTCATTGGGATCA
	β-Actin Forward (5' - 3')	TGTCCACCTTCCAGCAGATGT
	β-Actin Reverse (5' - 3')	AGCTCAGTAACAGTCCGCCTAG
Human	mtCOX-1 Forward (5' - 3')	ATGACCCACCAATCACATGC
	mtCOX-1 Reverse (5' - 3')	ATCACATGGCTAGGCCGGAG
	mtND1 Forward (5' - 3')	CATCACCCTCTACATCACCGCCCCG
	mtND1 Reverse (5' - 3')	TGAGTTTGATGCTCACCCTGATCAG
	mtND4 Forward (5' - 3')	TCCCTACAAATCTCCTTAATTATAA
	mtND4 Reverse (5' - 3')	GAAGGGAGCCTACTAGGGTGTAGAA
	Pai-1 Forward (5' - 3')	CCTGGGCACTTACAGGAAGG
	Pai-1 Reverse (5' - 3')	GGTCCGATTCGTCGTCAAATAAC
	TGF-β1 Forward (5' - 3')	GCAACAATTCCTGGCGATACCTC
	TGF-β1 Reverse (5' - 3')	CCTCCACGGCTCAACCACTG
	MCP-1 Forward (5' - 3')	CAGCAGCAAGTGTCCCAAAGAAG
	MCP-1 Reverse (5' - 3')	TGCTTGTCCAGGTGGTCCATG
	SARM1 Forward (5' - 3')	TGGGGACACTCCAGATGTCT
	SARM1 Reverse (5' - 3')	CTGCTTCCAGCTTCTCCACA
	β-Actin Forward (5' - 3')	CCACGAAACTACCTTCAACTCCATC
	β-Actin Reverse (5' - 3')	AGTGATCTCCTTCTGCATCCTGTC
